# Menopause in gynecologic cancer survivors: evidence for decision-making

**DOI:** 10.61622/rbgo/2025FPS1

**Published:** 2025-02-06

**Authors:** Agnaldo Lopes da Silva, Mariana Seabra Leite Praça, Rívia Mara Lamaita, Eduardo Batista Cândido, Lucia Helena Simões da Costa Paiva, José Maria Soares, Renato Moretti Marques, Maria Celeste Osório Wender

**Affiliations:** Universidade Federal de Minas Gerais Belo Horizonte MG Brazil Universidade Federal de Minas Gerais, Belo Horizonte, MG, Brazil; Universidade Federal de Minas Gerais Belo Horizonte MG Brazil Universidade Federal de Minas Gerais, Belo Horizonte, MG, Brazil; Universidade Federal de Minas Gerais Belo Horizonte MG Brazil Universidade Federal de Minas Gerais, Belo Horizonte, MG, Brazil; Universidade Federal de Minas Gerais Belo Horizonte MG Brazil Universidade Federal de Minas Gerais, Belo Horizonte, MG, Brazil; Universidade Estadual de Campinas Campinas SP Brazil Universidade Estadual de Campinas, Campinas, SP, Brazil; Faculdade de Medicina Universidade de São Paulo São Paulo SP Brazil Faculdade de Medicina, Universidade de São Paulo, São Paulo, SP, Brazil; Hospital Israelita Albert Einstein São Paulo SP Brazil Hospital Israelita Albert Einstein, São Paulo, SP, Brazil; Universidade Federal do Rio Grande do Sul Porto Alegre RS Brazil Universidade Federal do Rio Grande do Sul, Porto Alegre, RS, Brazil

## Abstract

• Although advances in the treatment of gynecological cancer have improved survival rates, they may also increase the effects of induced menopause, especially in young women.

• Cancer treatments such as oophorectomy, gonadotoxic chemotherapy, and pelvic radiotherapy can induce menopause.

• Gonadotoxic chemotherapy, especially alkylating-containing regimens, often damages ovarian function and may result in permanent menopause.

• Pelvic radiotherapy usually results in permanent loss of ovarian function unless ovarian transposition is performed.

• Diagnosing menopause after cancer is challenging, and common diagnostic criteria such as 12 months or more of amenorrhea and elevated follicle-stimulating hormone (FSH) levels are not entirely reliable, since ovarian function may return years after treatment.

• A multidisciplinary approach to post-cancer menopause is essential and should include an appropriate line of care, since hormone replacement therapy after treatment of gynecologic malignancy is controversial.

**Recommendations**

•An undetectable anti-Müllerian hormone for 30 months may suggest menopause in patients evaluated after breast cancer treatment.
•Although the primary goal is to achieve the best oncologic outcome, quality of life and long-term health outcomes should be considered.
•Ovarian preservation or ovarian transposition should be considered in selected cases of gynecological cancer.
•If hormone therapy (HT) is contraindicated, nonpharmacological interventions and nonhormonal treatments are available for vasomotor symptoms.
•Vaginal estrogen appears to be safe for most patients with gynecological cancer.
•Endometrial cancer: HT is indicated for symptomatic women after surgical treatment of early-stage endometrial cancer.
•Ovarian cancer: HT is indicated for high-grade serous carcinomas, clear cell carcinoma, mucinous carcinoma and borderline mucinous tumors.
•Hormone therapy may be considered in cases of cancer of the cervix, vulva and vagina.

## Background

Approximately 40% of women with gynecologic malignancies are premenopausal or perimenopausal at the time of diagnosis.^(1)^ Depending on the tumor type and staging, treatments may include hysterectomy with or without bilateral salpingo-oophorectomy, radiation therapy, and chemotherapy, resulting in loss of ovarian function. Survival is improving with new opportunities for cancer diagnosis and therapy, and consequently, more women are experiencing the long-term effects of certain types of treatments, such as loss of ovarian endocrine function.^(2)^ In women under 45 years of age, this can lead to induced menopause, increasing the risk of osteoporosis, cardiovascular disease, and cognitive decline. These negative effects of hypogonadism are related to the duration of the period of hypoestrogenism.^(3)^

Diagnosing menopause after cancer can be challenging, as menopausal symptoms may overlap with other common symptoms in cancer patients, such as fatigue and sexual dysfunction.^(2)^ The symptoms of induced menopause are more intense than those of physiological menopause due to the abrupt cessation of ovarian hormone production, especially estrogen, with a greater negative impact on quality of life.^(4)^ This phenomenon, combined with the physical symptoms and psychological problems related to cancer treatment, negatively impacts the quality of life of these women.^(3)^ In addition, advances in cancer treatment have increased patient survival, requiring monitoring of long-term risks associated with hypoestrogenism, such as osteoporosis and a higher incidence of cardiovascular disease.

Hormone therapy (HT) is the most effective treatment for the symptoms of ovarian failure and for the prevention of its late consequences in symptomatic women after treatment for gynecological cancer. However, in addition to the inherent risks of HT, there are concerns about its impact on recurrence of the treated cancer and patient survival. Management of menopausal symptoms in gynecologic cancer survivors should consider the woman’s age, tumor type and stage, use of antiestrogen therapies (for hormone-dependent cancers), and the presence of concomitant morbidities.^(3)^ When HT is contraindicated or avoided, emerging evidence supports the efficacy of nonpharmacologic interventions and nonhormonal pharmacologic treatments, although most of the evidence is based on women over 50 years of age with breast cancer.^(2)^

## What are the strategies for preserving ovarian function before cancer treatment?

Cancer treatments such as oophorectomy, gonadotoxic chemotherapy, and pelvic radiotherapy can induce menopause. Gonadotoxic chemotherapy, especially alkylating-containing regimens, often damages ovarian function and can lead to permanent menopause. There are few effective options for preventing ovarian damage caused by chemotherapy. The impact of new targeted therapies and immunotherapies on ovarian function is still uncertain, but studies in mice show that immunotherapy is gonadotoxic and reduces ovarian reserve.^(2)^

The effect of gonadotropin-releasing hormone (GnRH) analogues before chemotherapy at menopausal age and in long-term ovarian function is still unknown. Transposition of the ovaries out of the radiation field may be a protective measure and should be discussed as part of shared decision-making.^(2)^ This strategy prior to external pelvic radiation allows preservation of ovarian function in 20%-100% of cases, and the efficacy depends primarily on age.^(5)^

By recognizing the potential value of preserving ovarian function both in the short and long term, strategies to preserve fertility and ovarian function after cancer in premenopausal women are recommended in the following situations:^(2)^

**Figure 1:**
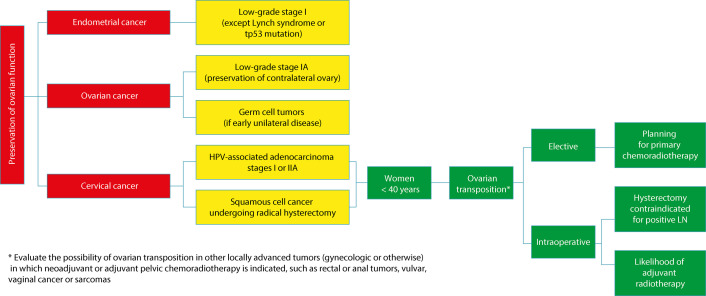
Definition of ovarian preservation according to gynecological cancer

Source: Adapted from Hickey et al. (2024).^(2)^

Consider ovarian preservation (Figure 1):

•Cervical cancer: human papillomavirus (HPV)-associated adenocarcinoma stages I or IIA and squamous cell carcinoma undergoing radical hysterectomy.
•Endometrial cancer: low-grade stage I (not associated with Lynch syndrome or TP53 mutation); future molecular subtypes of endometrial cancer may improve stratification of care.
•Ovarian cancer: low-grade stage IA (sparing the contralateral ovary; consider the 6%-13% risk of recurrence in the preserved ovary) and in germ cell tumors with early unilateral disease.
•Consider ovarian transposition in women younger than 40 years:
•Elective: women with locally advanced cervical cancer if primary chemoradiotherapy is planned.
•Intraoperative: women with cervical cancer in whom radical hysterectomy was aborted because of the presence of a positive lymph node (LN) or if adjuvant radiotherapy is likely.

## How to diagnose and manage menopause after cancer treatment?

Diagnosing menopause after cancer is challenging and there are no agreed criteria. Common diagnostic criteria, such as 12 months or more of amenorrhea and elevated follicle-stimulating hormone (FSH) levels, are not entirely reliable, as ovarian function may return years after treatment. Pelvic radiotherapy usually results in permanent loss of ovarian function unless ovarian transposition is performed. On the other hand, chemotherapy may allow recovery of ovarian function, especially in younger women. Although anti-Müllerian hormone may indicate reduced ovarian reserve after chemotherapy, it does not accurately predict fertility or the length of the reproductive period. However, an undetectable anti-Müllerian hormone at 30 months is a strong predictor of menopause after chemotherapy for breast cancer.^(2)^

Provided there are no contraindications, HT may be recommended to women under 45 years of age who have prolonged amenorrhea after gonadotoxic treatment. Treatment should be personalized, taking into account the patient’s age, type of cancer, time since diagnosis, quality of life (menopausal symptoms), comorbidities (such as venous thromboembolism [VTE], polypharmacy, and possible drug interactions), risk factors for chronic diseases (such as osteoporosis and ischemic heart disease), and patient preferences.

Since cancer patients are at high risk of VTE, and evidence suggests that transdermal HT does not significantly increase this risk, it is preferable to oral HT. Although the ideal duration of HT is uncertain, it should be considered until the average age of menopause (≥45 years), depending on symptoms and other health indicators, such as bone density.^(2)^

In addition to HT, non-hormonal pharmacological therapies and nonpharmacological interventions are important for the management of menopausal symptoms, expanded treatment options for vasomotor symptoms and, to a lesser extent, menopause-associated genitourinary symptoms. Shared decision-making including the participation of the patient and the oncologist responsible for the treatment should be adopted.^(2,4)^

## Cancer of the uterine body

Endometrial carcinoma (EC) is the sixth most common malignant tumor among women worldwide. Over the past 30 years, the incidence of these tumors has increased more than 130%, reflecting the increase in the prevalence of risk factors, especially obesity and population aging. Approximately ¼ of cases occur in the premenopausal period.^(6)^ In Brazil, there were 1,944 deaths related to this type of cancer in 2020.^(7)^ The vast majority of cases are diagnosed in their early stages, resulting in a good prognosis after surgical treatment, with or without radiotherapy. As most of these tumors express hormone receptors, there is reluctance to prescribe HT to survivors by the theoretical risk of promoting recurrence due to estrogen stimulation.^(8)^ The overall five-year survival rate for all stages is approximately 86%, and if the cancer is confined to the uterus, the five-year survival rate can increase to 97%.^(9)^

Type 1 endometrial cancer is the most common subtype, accounting for 90% of cases. It usually presents endometrioid histology with positive estrogen and progesterone receptors, and sensitivity to hormonal action. On the other hand, type 2 normally does not present hormone receptors or sensitivity to hormonal action, and its papillary serous histology has a more aggressive behavior and is diagnosed at a more advanced stage.^(10)^ The use of combined HT in cancer-free climacteric women reduces the risk of endometrial cancer, and the use of sequential combined HT does not increase this risk.^(4)^ In a 13-year follow-up of women participating in the Women’s Health Initiative (WHI), for example, a 33% reduction in the risk of endometrial cancer was observed with combined therapy.^(11)^ However, in women who have been treated for endometrial cancer, even after uterine removal, there is concern that the use of estrogen may stimulate hidden foci of tumor cells. In meta-analyses of retrospective studies, neither a higher risk of recurrence nor decreased survival have been observed.^(12)^

Although studies and guidelines suggest that HT may be an option to treat menopausal symptoms and prevent the long-term consequences of hypoestrogenism in women surviving early-stage endometrial cancer, these recommendations are based on low-quality evidence.

## Uterine sarcomas

Uterine sarcomas are a heterogeneous group of mesenchymal tumors and exhibit variable expression of hormone receptors. Although most cases are diagnosed after menopause, these tumors can occur in younger women. Immunohistochemical testing for estrogen and progesterone receptors may aid in decisions about HT. However, there are still no clinical trial data available to guide practice, even when hormone receptor expression is negative.^(3)^ Given the lack of studies on the use of HT in uterine sarcomas, it is not possible to determine the safety of its prescription in this group of tumors.^(12)^ To date, other non-hormonal strategies should be indicated to address menopausal symptoms and prevent and treat osteoporosis.^(12)^ The following guidelines are available for women survivors of endometrial cancer and uterine sarcoma:^(1-3,12-15)^

•Hormone therapy can be prescribed to symptomatic women after surgical treatment of early-stage endometrial cancer.
•There is no information available on the safety of HT in FIGO stages > II.
•There is no evidence to guide the use of HT in women with:
•High-grade non-estrogen-dependent endometrial cancer and carcinosarcoma
•Sarcomas: endometrial stromal sarcoma, leiomyosarcomas, and adenosarcomas
•Decision-making should be individualized and discussed in detail with patients. The woman’s symptoms and preferences should be taken into account.
•It is not possible to draw definitive conclusions about the type of hormone, method of administration, or duration of therapy.
•A valid strategy discussed in a multidisciplinary manner with clinical oncology is the evaluation of tumor immunohistochemical expression of hormone receptors. This enables an individualized approach in which the associated risks and benefits are considered.
•If adjuvant treatment is indicated in the management of these tumors, HT should be started at 6 to 12 months after treatment.

## Ovarian cancer

Ovarian cancer is the seventh most common cancer among women globally. In the general population, 1.4% of women will develop ovarian cancer and 1% of them will die from this condition.^(16)^ In Brazil, an estimated 7,310 new cases and around 3,920 deaths are caused by ovarian cancer each year.^(6)^ Most malignant ovarian tumors are diagnosed in advanced stages due to the lack of specific symptoms in the early stages. Despite much effort to identify an effective approach to screening for ovarian cancer, no test has yet been shown to reduce mortality from this neoplasm.^(15)^

One of the most widely used histological classifications for ovarian neoplasms is based on the embryonic origin of the cells. According to this classification, non-metastatic tumors can be epithelial, stromal and sex cord, or germ cell. As approximately 90% of ovarian cancers are of epithelial origin, this is the most frequent among malignant neoplasms. Cellular types include serous (30%-70%), mucinous (5%-20%), endometrioid (10%-20%), clear cell (3%-10%), and undifferentiated (1%). Serous carcinomas, the most common, account for 80%-85% of cases and are bilateral in up to 25% of cases. There are two types of serous carcinomas: low-grade and high-grade. Low-grade serous carcinoma progresses slowly and has a good prognosis, while high-grade serous carcinoma has an unfavorable course and is diagnosed in advanced stages. Borderline tumors, which may be serous or mucinous, account for 10% of epithelial ovarian tumors and have a better prognosis in most cases. Mucinous carcinomas account for less than 5% of cases, and are generally well differentiated and diagnosed in early stages, as are endometrioid and clear cell carcinomas.^(15)^

Even though several theories and many studies attempt to elucidate the relationships between cause and effect, the etiology of ovarian tumors remains unknown. The origin of ovarian neoplasms is believed to be associated with a set of factors, such as environmental, reproductive, dietary and infectious factors, exposure to teratogenic agents and genetic and endocrine issues. Data are scarce and have not provided convincing evidence on the role of estrogen in ovarian carcinogenesis. Current and recent use of hormonal therapy is associated with a small but statistically significant risk of ovarian cancer in observational studies, especially for serous cancer.^(4)^

Evidence suggests better overall survival in women with operated epithelial ovarian cancer who receive HT, compared to those who do not use HT, although the certainty of the evidence is low. In general, the results of the studies show no negative effect of HT on the risk of recurrence. However, data did not evaluate definitive results regarding the various histological subgroups or the characterization of ovarian cancer.^(14,17,18)^ Women treated for ovarian cancer who present symptoms and worsening quality of life should receive specialized follow-up to evaluate the indication for HT. Some guidelines on HT in this situation are:^(1,12,14,15,18)^

•Hormone therapy can be initiated after completion of treatment and should not be prescribed concomitantly with biological or experimental treatments for ovarian cancer.
•As high-grade serous carcinomas are not predominantly estrogen-dependent, HT can be used.
•As low-grade serous carcinomas may express estrogen and progesterone receptors, i.e., are hormone-dependent, HT is not recommended, and non-hormonal alternatives should be considered.
•In endometrioid carcinomas, especially with positive hormone expression, great caution is needed when considering HT.
•Hormone therapy can be prescribed in mucinous carcinomas.
•In clear cell carcinomas, due to the increased risk of VTE, transdermal administration of HT is recommended.
•As borderline serous tumors can progress to low-grade serous tumors, which are hormone-responsive, administering HT requires caution.
•Hormone therapy may be administered in borderline mucinous tumors without risk factors.
•Stromal tumors are hormone-dependent in nature and can be treated with anti-endocrine therapy. Therefore, HT is not recommended due to the risk of stimulating tumor growth.
•Germ cell tumors usually affect young patients and can be treated with fertility-preserving surgery. Hormone therapy may be used to alleviate menopausal symptoms and improve the quality of life of patients.
•Routine assessments of hormone receptor status may help to stratify patients into low- and high-risk groups for HT.
•These considerations may refer to a short course of HT (2-5 years). In cases of iatrogenic early menopause, a multidisciplinary approach should be implemented to decide on continuation of HT use for a longer period.

## Cervical cancer

Cervical cancer is the fourth most common cancer among women globally. In Brazil, the expected annual incidence is 17,010 new cases between 2023 and 2025, making it the third most common cancer among Brazilian women.^(6)^

There are no data demonstrating the effect of HT on the recurrence rate or cancer outcomes in patients with cervical squamous cell carcinoma or adenocarcinoma. Hormone therapy varies according to the treatment of the cervix. In patients treated surgically with radical hysterectomy, estrogen-only therapy is recommended. For patients undergoing chemoradiation, continuous combination therapy is proposed. Considerations for HT in women with cervical cancer are as follows:^(2,14,19)^

•Hormone therapy should be considered for treatment-related menopause.
•The therapy regimen (unopposed estrogen or estrogen plus progestin) depends on the presence or absence of a uterus.
•Therapy may be prescribed for both early-stage patients undergoing surgery and locally advanced patients receiving chemoradiation alone.
•The use of moisturizers or vaginal estrogen after radiation is recommended. Vaginal dilators may be required.

## Vulvar and vaginal cancer

Cancers of the vulva and vagina are rare and, when combined, account for less than 10% of all gynecological cancers.^(6)^ At the time of diagnosis, most affected women are postmenopausal. Squamous cell carcinoma is the most common type in both cases and often related to HPV infection. Similar to cervical cancer, squamous cell carcinomas of the vulva and vagina are not hormone-dependent. There is no evidence that the use of HT after treatment of these cancers is associated with a higher recurrence rate or lower survival.^(20)^ In women under 40 years of age, premenopausal and with an indication for pelvic chemoradiotherapy, ovarian transposition should be considered.^(1,21)^

## Women with BRCA1 and BRCA2 mutations undergoing risk-reducing salpingo-oophorectomy

The lifetime risk of carriers of germline pathogenic variants in BRCA1 and BRCA2 developing ovarian cancer is approximately 44% and of developing breast cancer is 72% to 69%. Risk-reducing salpingo-oophorectomy (RRSO) can significantly reduce these risks. Women and clinicians considering RRSO should be aware of these risks, and clinical care should focus on safe options available for symptom management and long-term health optimization.^(4,21)^ In these patients, HT can both improve quality of life and increase life expectancy without negating the protective effects of RRSO and without increasing subsequent risk of breast cancer.^(1,12)^ Hormone therapy recommendations for women with no history of breast cancer undergoing RRSOR:^(12,21)^

•Hormone therapy after RRSO for women up to 45 years of age: indicated for relief of vasomotor symptoms and/or disease prevention.
•Hormone therapy after 45 years of age: should be individualized, considering the need for progestin, prior risk-reducing mastectomy, symptom severity, and risk factors for osteoporosis.
•Transdermal HT is recommended for women at high risk of VTE.

## Women with Lynch syndrome undergoing hysterectomy with risk-reducing salpingo-oophorectomy(3,22)

•In patients with a history of Lynch syndrome who carry germline mutations in the DNA repair genes – MSH2, MLH1 and MSH6 –, hysterectomy with RRSO is indicated between the ages of 35 and 40. Estrogen-only HT may be considered in these women for relief of climacteric symptoms.
•In this specific group, there is also the benefit of reducing the incidence of colorectal cancer, and HT may be recommended.
•In patients with a history of previous colorectal cancer, data in the literature show improvement in related outcomes.
•Due to the absence of a significant increase in ovarian cancer, carriers of mutations in the PMS2 gene may avoid bilateral salpingo-oophorectomy.

## What are the non-hormonal therapies for the treatment of climacteric symptoms in survivors of gynecological cancer?

For patients in whom HT is contraindicated or should be avoided after cancer, a growing number of nonhormonal therapies are available to treat vasomotor symptoms. These nonpharmacological interventions appear to have some efficacy in controlling vasomotor symptoms. Lifestyle modifications, relaxation training, and mindfulness-based stress reduction are viable options that also improve sleep, depressive symptoms, and self-reported stress. Cognitive behavioral therapy and other therapeutic practices such as hypnosis, stellate ganglion blockade, acupuncture, and yoga have also shown benefits, including in patients after cancer treatment.^(2,3)^

However, unlike HT, other non-hormonal drug therapies do not improve genitourinary symptoms nor prevent fractures. Anticonvulsants such as gabapentin have similar efficacy, while the antihypertensive clonidine is less effective and currently not recommended. Targeted therapy with the neurokinin B receptor antagonist fezolinetant is a new pharmacological option and already available in some countries, but has not yet been evaluated in cancer patients. Its use for the treatment of vasomotor symptoms has shown a significant reduction in the frequency and severity of hot flashes, with an impact on quality of life.^(2)^ Non-hormonal treatments should be selected on a shared decision-making approach. A suggested approach to initiating these treatments includes the use of paroxetine 7.5 to 20 mg, venlafaxine 37.5 to 150 mg, desvenlafaxine 100 to 150 mg, escitalopram or citalopram 10-20 mg. It is recommended to start with lower doses and gradually increase according to clinical response. Oxybutynin 2.5 to 5 mg has also been used, but has more adverse effects, such as cognitive decline with prolonged use. Gabapentin (300-2,400 mg) reduces night sweats and causes drowsiness, which may improve sleep.^(2,3)^ The dose of 900 mg divided into three doses of 300 mg/day is the most commonly used. Data with pregabalin are more limited and due to the potential for abusive use, is not recommended.

Sexual issues should be addressed and discussed by health professionals with all cancer patients since the beginning of treatment. Psychosocial or psychosexual counseling should be offered to improve sexual response, body image, intimacy and relationship issues, as well as overall sexual functioning and satisfaction.

## What are the possible treatments for genitourinary syndrome of menopause in gynecological cancer survivors?

The treatment of genitourinary syndrome (GUS) and sexual dysfunction recommends non-hormonal agents as first-line therapy, reserving vaginal estrogens for persistent symptoms. However, vaginal estrogens are still the best treatments for this condition. They favor the reestablishment of vulvovaginal trophism, promoting improvement in vaginal dryness with no significant differences between products. Promestriene, 17-β-estradiol and estriol are among the options available in Brazil for vaginal use. The recommendation for use of any of these formulations is an intravaginal application at night for 14 days, followed by a maintenance application two to three times a week while symptoms persist. Improvement in GUS generally occurs from the third week after starting local therapy (level of evidence A), although some women require approximately 12 weeks or more of treatment to obtain optimal results. It is known that the responsiveness to treatment is lower with increasing age, so administration should be started as soon as symptoms resulting from atrophy begin. There is no age limit for starting treatment.

Since there is little evidence from clinical trials to support the efficacy of lubricants and moisturizers, in most guidelines the use of topical estrogen is considered superior to vaginal moisturizers, and is the standard treatment for GUS.^(2,22,23)^

There is also the possibility of using energy-based therapies to treat genitourinary symptoms in these patients. Laser therapy can be considered a therapeutic option that allows women to avoid hormonal interventions in the treatment of GUS. Microablative fractional CO_2_ laser or non-ablative vaginal Erbium YAG laser (VEL) can be used. Treatment with CO_2_ or VEL laser generally consists of a series of three to four applications at four to six weeks intervals on an outpatient basis. Although there are studies suggesting an improvement in vaginal health after the use of energy-based therapies, most of them were not controlled by simulation or placebo and included a small number of patients without cancer and a short follow-up period.^(22)^ Therefore, laser therapy should be indicated and used with caution or as a potential alternative in the treatment of GUS. Failure to respond satisfactorily to the use of vaginal estrogens, contraindications to the use of HT or refusal to use vaginal estrogens may be possible indications. Given the lack of studies on the safety of energy-based therapies in patients treated for cervical cancer, they are not recommended.

**Figure 2:**
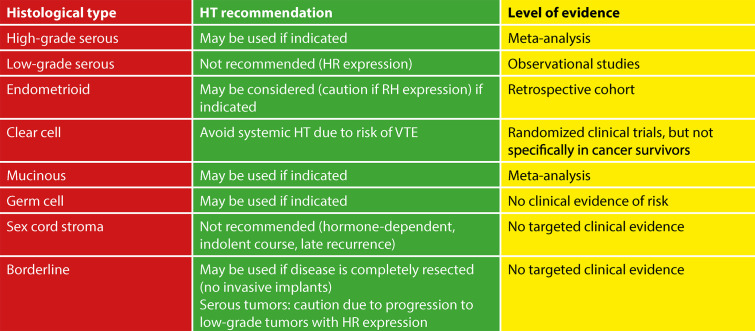
Recommendations for hormone replacement therapy in ovarian cancer by histological type

**Figure 3:**
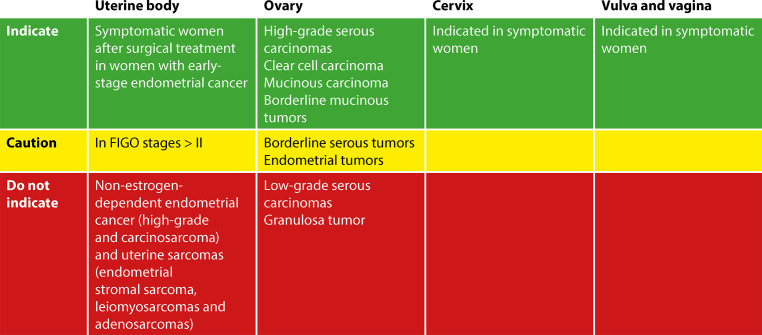
Proposal for clinical decision-making on the indication of hormone therapy for women survivors of gynecological cancer TTO - treatment Note: Hormone therapy may be prescribed for symptomatic women who do not improve with non-hormonal therapies, on an individualized basis, considering the woman’s symptoms and preferences.

**Source:** Adapted from The 2022 Hormone Therapy Position Statement of The North American Menopause Society” Advisory Panel.^(12)^

The vaginal effects of external beam radiation therapy or brachytherapy, especially vaginal dryness, fibrosis, and shortening, which can cause discomfort or pain during sexual activity and impair sexual function, may be more severe than those of chemotherapy. Vaginal dilators are recommended to maintain vaginal function after pelvic radiation. However, adherence is low - less than 25% of patients use them - and the evidence base is limited. Vaginal estrogen is commonly offered, although there is little evidence to support its efficacy.^(2,22)^ Multidisciplinary evaluation and follow-up of these patients with the involvement of physical therapists trained in pelvic floor assessment after gynecologic cancer treatment is extremely important and should be indicated when available (Figures 2 and 3).

HT - hormone therapy; HR - hormone receptors; VTE - venous thromboembolism Source: Adapted from Brennan et al. (2021).^(22)^

## Final considerations

Advances in early detection and treatment of gynecological malignancies have improved patient survival. However, these gains are often accompanied by treatment-associated toxicities that negatively affect quality of life. The management of climacteric symptoms should follow a shared decision-making approach, taking into account the patient’s age, type of cancer, time since diagnosis, quality of life, comorbidities, and personal preferences. Hormone therapy is effective in treating vasomotor symptoms and appears to be safe for many gynecologic cancer survivors. Although most evidence suggests a benefit of HT in gynecologic cancer survivors, both in terms of quality of life and, in some cases, in improving overall survival, HT is still underprescribed overall. The underuse of HT is likely multifactorial, with concerns among patients and physicians about adverse oncologic outcomes, including the risk of recurrence. Nonpharmacological interventions are emerging as effective alternatives, expanding the treatment options for vasomotor symptoms and genitourinary symptoms associated with induced menopause.
